# Accuracy of Zygomatic Implant Placement Using Completely Versus Partially Guided

**DOI:** 10.1186/s12903-026-08436-x

**Published:** 2026-05-06

**Authors:** Mohamed Kamal Ghallab, Alshaimaa Ahmed Shabaan, Haytham Al-Mahalawy, Moustafa Taha

**Affiliations:** 1 Oral & Maxillofacial Surgery Department, Faculty of Dentistry, Fayoum University, Fayoum, Egypt; 2 Oral & Maxillofacial Surgery Department, Faculty of Dentistry, Pharos University, Alexandria, Egypt; 3 Oral & Maxillofacial Surgery Department, Faculty of Dentistry, Ain Shams University, Cairo, Egypt

**Keywords:** Zygomatic implant, atrophic maxilla, computer guided surgery, surgical guide

## Abstract

**Objective:**

Zygomatic implant placement is a challenging surgical procedure, as the implants are inserted with limited accessibility and visibility. The aim of this study was to compare the accuracy of completely limiting versus partially limiting computer-generated 3D surgical guides for zygomatic implant placement in patients with atrophic maxillae.

**Materials and methods:**

This prospective randomized controlled study included participants with atrophic posterior maxilla indicated for zygomatic implant (ZI) placement. The participants were randomly allocated to one of two groups. Group I received ZI using completely limiting 3D computer-guided surgical templates, while Group II underwent ZI placement using partially limiting 3D computer-guided surgical templates. The primary outcome variable was the accuracy of implant placement, which was assessed by measuring linear deviations at the implant coronal and apical points, as well as angular deviations. The secondary outcome was the duration of the operative time.

**Results:**

This study evaluated 12 zygomatic implants (6 per group) in 6 patients (mean age: 56.9 ± 7.57 years). Baseline demographics and clinical features were well-balanced between groups, with comparable age and sex distributions. There were no statistically significant differences in linear or angular deviations between the groups, except for a statistically significant greater apical deviation in the midsagittal plane for Group II (*p* = 0.015). Group II had a significantly shorter mean operative time (21.17 ± 2.14 min) than Group I (27 ± 2.37 min) (*p* = 0.01). No dropouts or postoperative complications (e.g., infection, peri-implantitis, or sinus-related issues) have occurred.

**Conclusions:**

Within the limitations of the sample size of this study, the partially limiting surgical guide could provide a successful and less invasive treatment modality for zygomatic implant placement, as it significantly reduces the surgical time with comparable overall accuracy. However, the completely limiting guide demonstrates superior control at the critical apical implant position.

**Trial registration:**

ClinicalTrials.org (NCT06227351) 2024-02-05.

**Supplementary Information:**

The online version contains supplementary material available at 10.1186/s12903-026-08436-x.

## Introduction

Zygomatic implants (ZIs) have provided a viable option for the immediate rehabilitation of the atrophic maxilla. The use of ZIs reduces treatment time while demonstrating favorable functional outcomes and patient satisfaction [1–3]. ZI surgery is a challenging procedure, as these long implants are inserted with limited accessibility and visibility. Moreover, the long drills used during the surgery could be difficult to control, and improper surgical techniques may lead to damage of vital structures or compromised implant positions that result in compromised final prosthesis [4–7].

Achieving precise ZI positioning and angulation is critical. Digital technologies—including implant planning software, cone-beam computed tomography (CBCT), and desktop 3D printers—have been employed to enhance the safety and predictability of ZI surgery [8, 9]. To further improve the accuracy of computer-aided ZI surgery, several modifications to the drilling guide have been implemented, aiming to minimize deviations from the planned position [10–12].

Surgical guides include static and dynamic techniques. A static surgical guide is commonly employed in implant placement. It includes complete limiting and partially limiting guides. The complete limiting surgical guide ensures complete guidance for both osteotomy and implant placement, while the partial limiting surgical guide provides guidance for the pilot osteotomy, with subsequent osteotomy expansion and implant insertion performed freehand [13, 14].

The complete limiting surgical guide provides an accurate definition of implant position and reduces the effect of the surgeon’s experience on ZI placement, but the field of vision during surgery is very limited, which could cause a minor deviation. However, the partial limiting guides offer benefits such as controlled irrigation, better accessibility for patients with limited mouth opening, and the ability to manually adjust the implant’s angulation or position during the procedure [13, 15, 16].

Current literature demonstrates limited evidence supporting the use of partially guided surgery for zygomatic implant placement. Moreover, no studies provide strong evidence about an optimum degree of accuracy. Wang et al. [17] recommended more randomized controlled clinical trials to further compare the differences between the guided protocols. The aim of this study was to evaluate the accuracy of completely limiting versus partially limiting computer-generated 3D surgical guides for zygomatic implant placement in patients with atrophic maxillae.

## Materials and methods

This study was conducted between March 2024 and December 2024 at the Oral and Maxillofacial Department, Faculty of Dentistry, Fayoum University. The study was approved by Fayoum university supreme committee for scientific research ethics (FU-SCSRE) (code: 102317) and the protocol was registered on ClinicalTrials.org (ID: NCT06227351) in February 2024.

All patients gave informed consent for the scientific use of their clinical data and images before the study began. The study was conducted in compliance with the Declaration of Helsinki and reported following the CONSORT guidelines 2025 [18].

### Study design & randomization

The study was a prospective, randomized (1:1), blinded clinical trial. Simple randomization was used to ensure balanced allocation of participants between groups. Predefined computer-generated random codes were sealed in opaque envelopes, with treatment allocation performed by an independent operator not involved in the study. The assessor was blinded to the patient group allocation and the type of surgical guide used. The primary CBCT data and superimposed models were anonymized and presented without any identifiers linking them to the surgical protocol.

### Participants

This study included patients with atrophic posterior maxilla. The inclusion criteria were as follows: (1) Patients > 18 years; (2) Patients with residual alveolar ridge height of < 4 mm in the region distal to the canine pillar. The exclusion criteria were (1) patients with conditions contraindicating implant placement, (2) patients with acute maxillary sinus infection or maxillary sinus cyst and (3) restricted mouth opening (less than 3 cm inter-arch distance anteriorly). Patients were randomly allocated to one of two treatment groups as follows: Group I received ZI using completely limiting 3D computer-guided surgical templates, while Group II underwent ZI using partially limiting 3D computer-guided surgical templates. All patients underwent a thorough preoperative clinical and radiographic examination using CBCT (Planmeca Promax 3D classic, Planmeca, Finland) with standard settings (90 kV, 6.3 mA, exposure period, 12 s, and voxel size, 0.2 mm). All participants in both groups were completely edentulous in the maxilla and had opposing natural mandibular dentition.

### Sample size estimation

Based on data from a previous study [8] (the mean angular deviations: 1.19° in Group I vs. 4.92° in Group II), a sample size calculation using G*Power 3.1.9.4 was performed with 95% power, α = 0.05, and an effect size of 3.003. This analysis indicated a requirement of 10 implants. To compensate for potential dropouts, the study included 12 implants (6 per group).

### Implant and surgical guide planning

The preoperative Digital Imaging and communication in Medicine (DICOM) files were obtained from CBCT and imported into the BlueSky-bio software (version 4.13.1). The data were segmented, and a three-dimensional reconstruction of the relevant anatomical structures was performed. The alveolar crest at the planned implant site was analyzed in three dimensions to assess bone height, width, and morphology relative to the prosthetic target. Based on this analysis, each site was preoperatively classified according to the Zygoma Anatomy Guided Approach (ZAGA) protocol. A three-step approach was employed to determine optimal implant placement in accordance with the ZAGA guidelines [9, 19]. The ZAGA classification was determined preoperatively based on the CBCT analysis of the alveolar ridge, zygomatic bone and maxillary sinus anatomy. First, the ideal coronal position of the implant head was identified. Next, the entry point on the zygomatic bone was selected based on anatomical suitability and biomechanical stability. Finally, these two points were connected to establishing the definitive implant trajectory, ensuring an optimal path while avoiding critical anatomical structures.

**Fig. 1:**
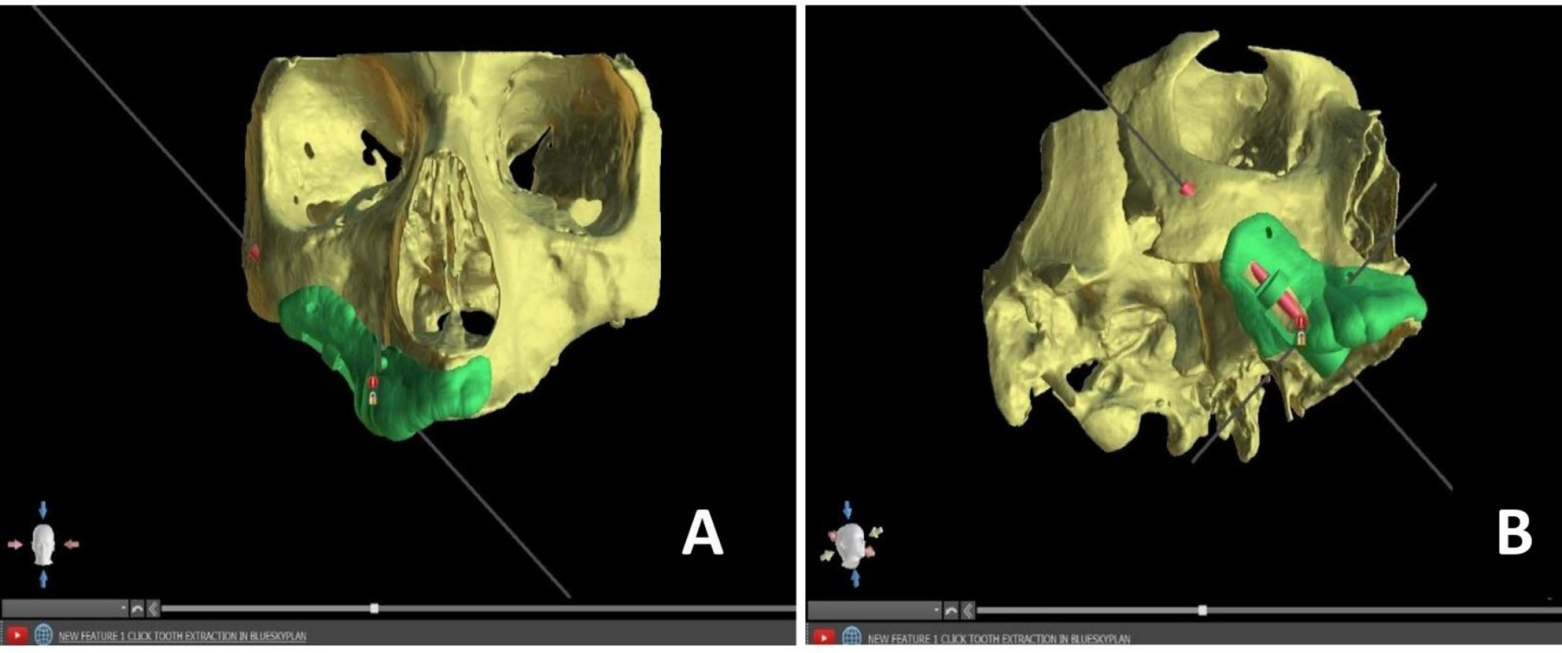
**A** Virtual surgical guide (frontal view), **B** Virtual surgical guide (sideview)

All planned ZI had sufficient bone coverage surrounding the implant threads. The virtual implants were enveloped by at least 3 mm of bone at the zygomatic bone level. The virtual surgical guide was created using Blue Sky Plan software. A cut-off window was incorporated into the surgical guide, aligning with its trajectory (Fig. 1). The surgical guide was exported into STL format. The exported file was 3D-printed from 405 nm UV resin (Proshape Digital Solutions) using a 4k EPAX 3D printer machine. Metal sleeves corresponding to the diameter of the shank of zygomatic implant drills were fixed in the guides. Before surgery, the guides underwent high-level disinfection by immersion in 2.4% activated glutaraldehyde solution for 20–30 min, followed by rinsing with sterile saline.

### Surgical procedures

For all patients, the same surgeon performed all the virtual 3D planning and the surgical interventions. Procedures were uniformly conducted under general anesthesia, with preoperative site preparation adhering to institutional operating room standards. After incision, reflection of the mucoperiosteal flap, and exposure of all relevant anatomical landmarks, the surgical templates were positioned using the anterior nasal spine as a guide for seating. Once the templates were properly seated, they were secured in place using 2.0 mm diameter screws ranging from 10 to 14 mm through 3 fixation points to ensure maximum stability and minimize micro-movement during drilling. Objective verification of the stability and seating of the surgical guide before and during drilling was performed via manual stability check and was double-checked and confirmed by a second assessor. The osteotomy drilling process was carried out in two distinct steps. Initially, crestal preparation was done sequentially using burs from the Universal Densah^®^ Bur Kit. Sequential drills were inserted palatally into the maxilla, passing through the alveolar crest and extending to the buccal side. The alveolar osteotomy was finalized using a drill with a diameter equivalent to the shank of zygomatic drills in the manufacturer’s zygomatic implant drilling kit.

All the drilling steps of the crestal preparation were done through metal keys with different diameters that fit into the metal sleeves in the surgical templates. After crestal osteotomy, a zygomatic diamond bur was used through the coronal and apical sleeves directly for groove preparation for leading the zygomatic implant drills.

**Fig. 2:**
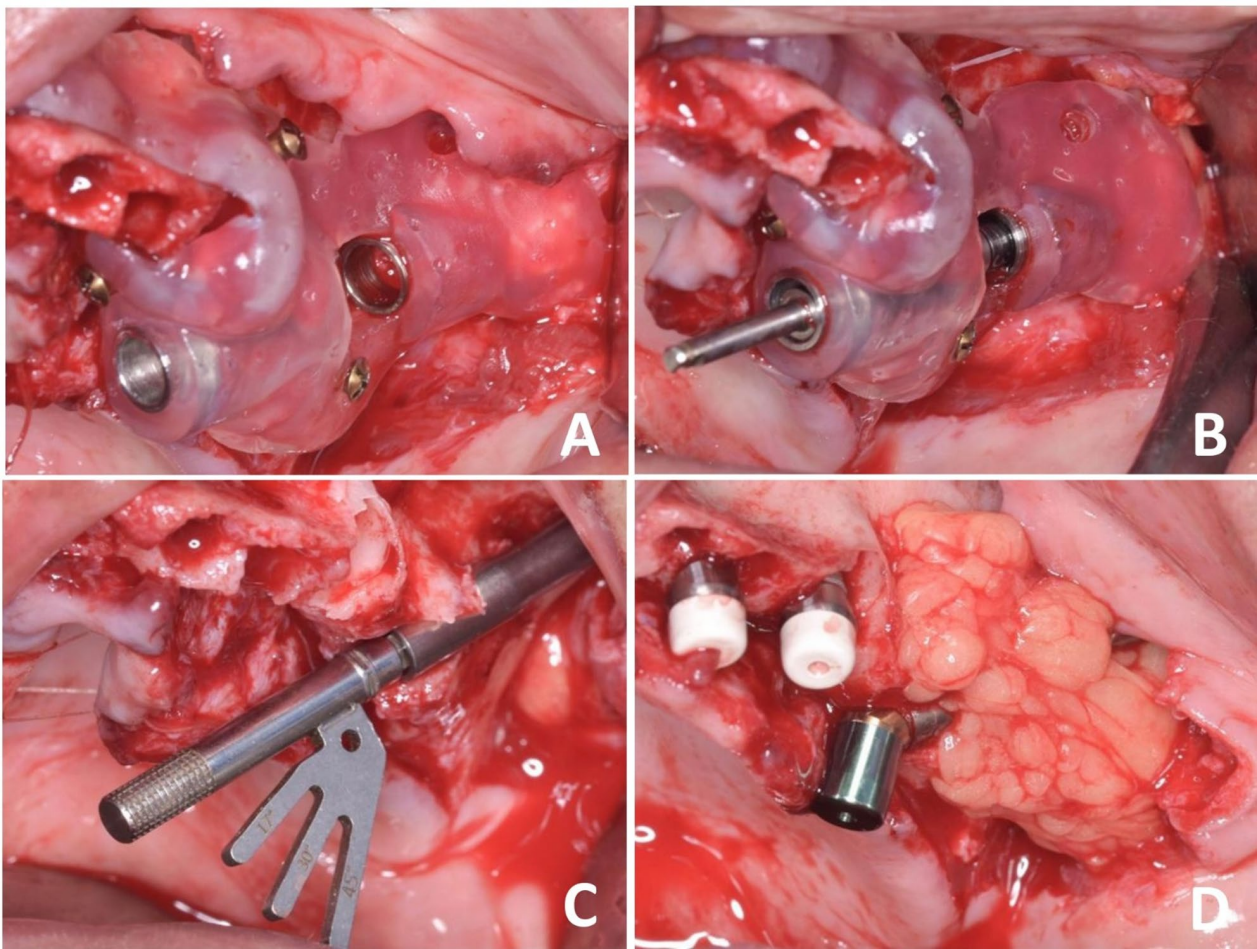
In the complete limiting group, **A** shows the fully seated left surgical guide, **B** illustrates the drill is properly fitted into the coronal and apical sleeves, **C** placement of the left-side zygomatic implant and determination of the multi-unit angle, **D** covering the left zygomatic implant with buccal pad of fat

**Fig. 3:**
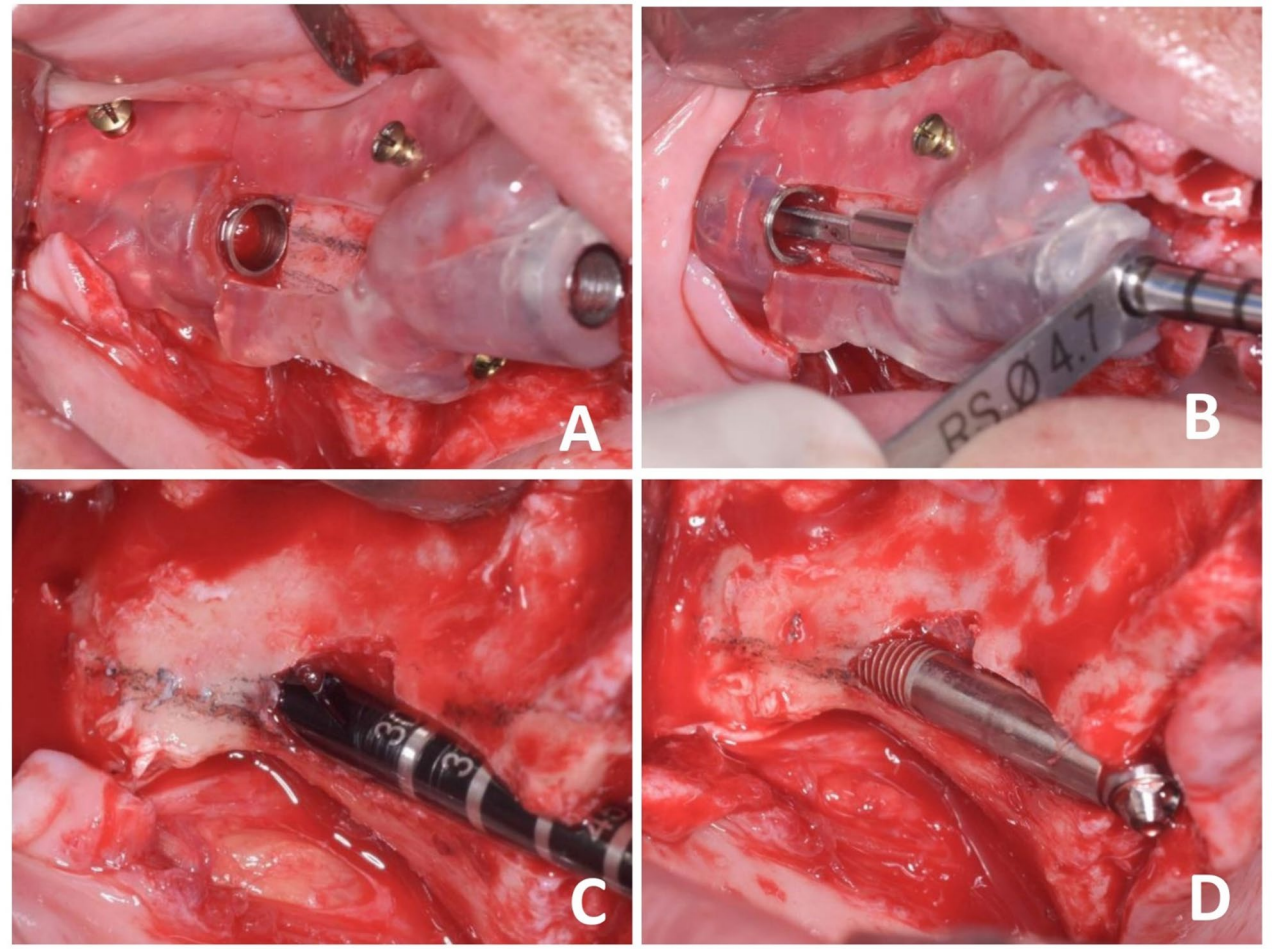
In the partially limiting group, **A** shows the fully seated right surgical guide, **B** illustrates drilling through the key, **C** shows removing the guide and continuously freehand drilling with drill no. 2, **D** shows placement of the right-side zygomatic implant

In group I, three drills of the zygomatic kit were used through the sleeves to enter the zygomatic bone (Fig. 2). While in group II, only the first drill of the zygomatic drills was used; then the guide was removed, and the remaining osteotomy and implant placement were completed freehand (Fig. 3).

Once the osteotomies were fully completed and the surgical guides removed, a depth gauge was employed to verify the depth and direction of the osteotomy and confirm the appropriate zygomatic implant length. The zygomatic implants were then manually inserted, ensuring the insertion torque did not exceed 80 N/cm, until the implant tip reached the inferior border of the zygomatic osteotomy exit point.

Operative time was defined as the duration from the initial surgical guide placement to the final positioning of the zygomatic implant. This measurement was recorded for both groups.

Then, the surgical wound was irrigated by sterile saline. For enhancing soft tissue quality, buccal pads of fat were used to cover the implant’s extra bony shafts. The wound was then closed using 4 − 0 vicryl sutures.

### Postoperative deviation assessment

**Fig. 4:**
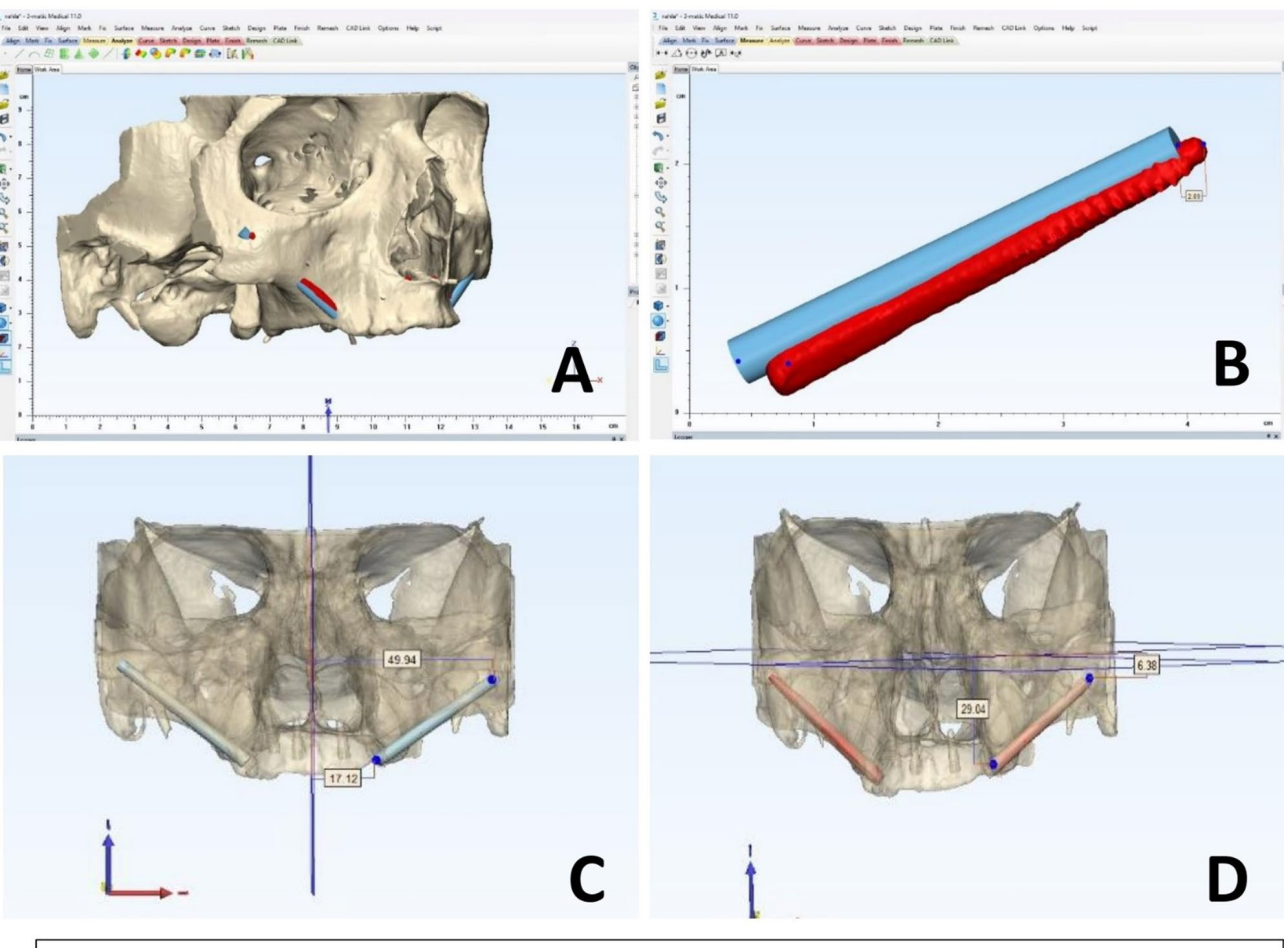
**A** superimposition of the planned virtual implant (blue) with the actual postoperative implant (red), **B** measuring the distance between the apical points of the planned and actual implants, **C** measuring the coronal and apical points of the virtual implant to the mid-sagittal plane, **D** measuring the coronal and apical points of the actual implant to the Frankfort horizontal plane

All patients underwent scheduled postoperative assessments: an initial evaluation at 4–5 days to verify proper healing and rule out infection, followed by suture removal at the 10-day visit once wound closure was confirmed. Postoperative implant positions were radiographically assessed using CBCT scans, performed under the same parameters and at the same radiology center as the preoperative scans. The assessor of the postoperative implant position was blinded to the group allocation. The preoperative virtual implant plans and actual postoperative 3D implant models were segmented, extracted, and superimposed using BlueSky Bio version 4.13 and 3-matic Medical 14.0 software. Anatomical landmarks—including the infraorbital foramina, anterior nasal spine, posterior nasal spine, and skull base or vault structures—were used for registration. Following superimposition (Fig. 4), fixed reference points were marked at identical locations on both the planned and actual implants, including coronal and apical points. Spatial analysis was conducted relative to three anatomical planes: the mid-sagittal plane (MSP) defined by the anterior nasal spine, nasion, and basion; the axial Frankfort horizontal plane (FHP) aligned with the porion and orbitale; and the coronal plane (CP) perpendicular to both MSP and FHP. Deviations were quantified by measuring linear distances from implant coronal and apical points to each plane, as well as the direct 3D displacement between corresponding virtual and postoperative implant points. Angular deviation was calculated as the difference between the long axes of planned and placed implants. All measurements were performed by a single, calibrated examiner. To determine intra-observer reliability, the entire measurement protocol was repeated on the same dataset after a one-week interval.

### Statistical analysis

The implants were considered the unit of analysis. The consistency of the repeated measurements (intra-observer reliability) was evaluated using the Intraclass Correlation Coefficient (ICC) based on a two-way mixed-effects and absolute-agreement model. The resulting ICC value of 0.98 (95% CI: 0.96–0.99) indicated excellent reliability. All analyses were performed using R (v4.4.1) with the Tidyverse, psych, and doBy packages. Categorical variables are presented as frequencies (percentages), while continuous variables are expressed as mean ± SD (normally distributed) or median (range) (non-normal). Normality was assessed using Shapiro-Wilk tests. Intergroup comparisons employed Welch’s t-tests (parametric continuous data), Mann-Whitney U tests (non-parametric continuous data), and Fisher’s exact tests (categorical variables: sex, side). Statistical significance was set at *p* ≤ 0.05.

## Results

### Participant data

This study involved a total of 12 implants (6 per group) and included 6 patients (4 males and 2 females). The mean patient’s age was 56.9 ± 7.57 years. A comparison of the two groups is presented in Table 1, outlining the demographic and clinical characteristics. The analysis showed no statistically significant differences in age or gender distribution between the groups.

**Table 1:** Demographic characteristics of patients in complete and partial limiting groups (*N* = 12)

Variable	Group I (Complete Limiting) *n* = 6 implants mean ± SD	Group II (Partial Limiting) *n* = 6 implants mean ± SD	*p*-value
Age (Years)	53.17 ± 9.17	60.67 ± 4.41	0.113
Sex n (%)			
Male	4(66.7%)	0(0%)	0.061
Female	2(33.3%)	6(100%)	
Side n (%)			
Right	3(50%)	2(33.3%)	1
Left	3(50%)	4(66.7%)	
ZAGA classification n (%)			
ZAGA 2	4(66.7%)	6 (100%)	0.061
ZAGA 3	2 (33.3%)	0 (0%)	

* Significant (*p*-value < 0.05.); ZAGA= Zygoma Anatomy Guided Approach

**Fig. 5:**
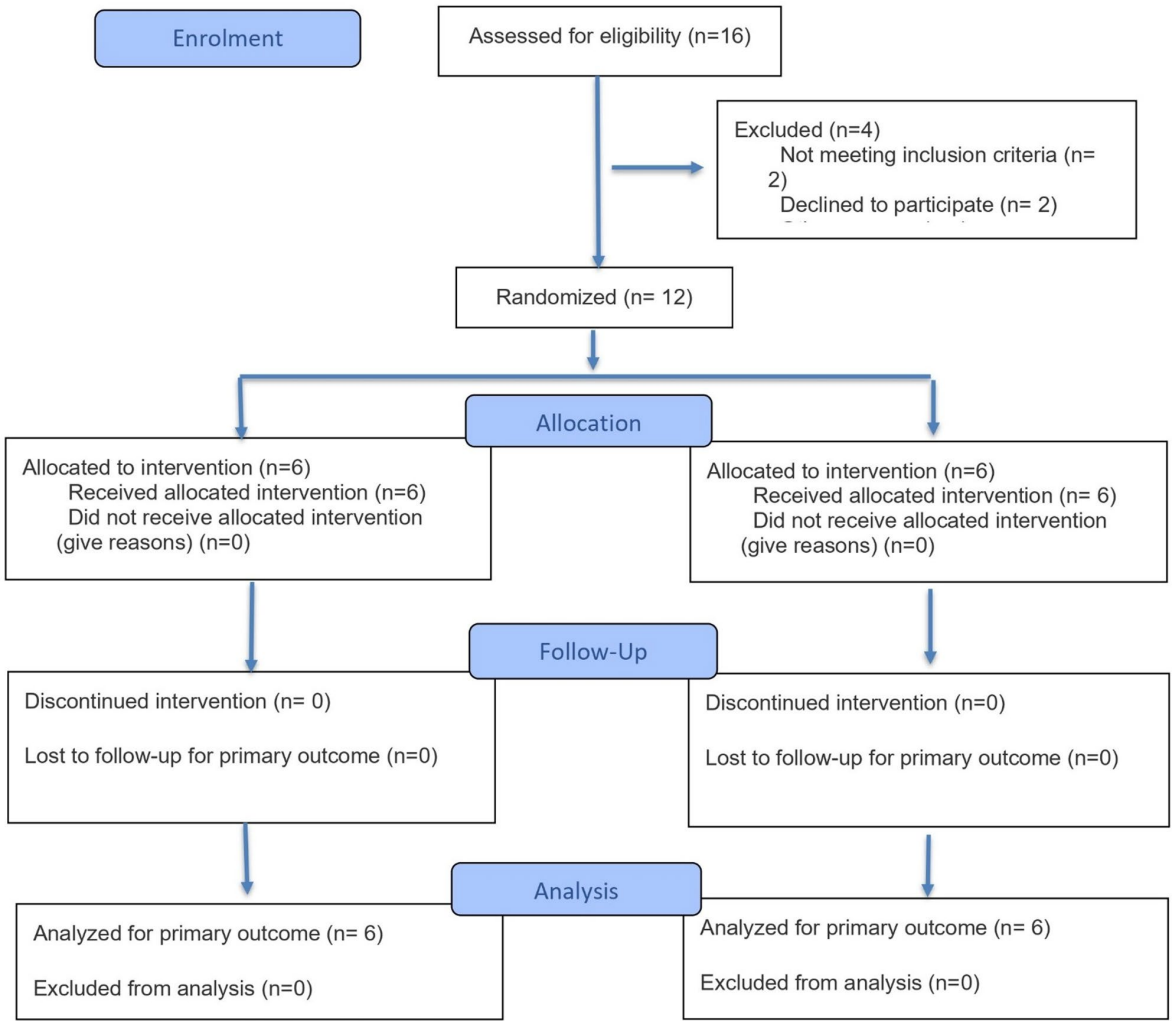
Consort flowchart diagram

All participants in both groups completed the study without attrition (Fig. 5). Furthermore, no postoperative complications linked to infection were reported. Healing progressed smoothly in all instances, with no indications of peri-implantitis observed. Post-zygomatic implant placement examinations conducted at the 4–5 day and 10-day follow-up visits revealed that all patients were free from clinical symptoms of sinus disruption, such as nasal congestion, nasal discharge, headaches, or oroantral communication. All participants in both groups were completely edentulous in the maxilla and had opposing natural mandibular dentition.

The mean operative time was significantly shorter in Group II (21.17 ± 2.14 min) compared to Group I (27 ± 2.37 min), with a statistically significant difference (*p* = 0.01).

### Postoperative deviation assessment

The Linear and angular deviations showed no statistically significant differences between the two groups in either linear measurements or angular deviations in implant placement (Table 2).

**Table 2:** Linear and angular deviations between planned and actual implant positions

Variable	Group I (Complete Limiting) *n* = 6 implants	Group II (Partial Limiting) *n* = 6 implants	*p*-value
Deviation distance at coronal point (mm)	1.89 ± 0.961	2.52 ± 1.25	0.356
Distance deviation at apical point (mm)	2.58 (2.14 − 4.18)	2.67 (2.1 − 7.98)	0.8182
Angular deviation between planned and actual implant positions	2.53 (0.55 − 4.71)	2.46 (1.59 − 8.02)	1

* Significant (*p*-value < 0.05.)

The coronal point measurements relative to different anatomical planes showed no statistically significant differences between the two groups for all the coronal measurements (Table 3).

**Table 3:** Linear deviations at the coronal point relative to the anatomical planes (CP, MSH, FHP) in the study groups

Variable	Group I (Complete Limiting) *n* = 6 implants	Group II (Partial Limiting) *n* = 6 implants	*p*-value
Planned MSP (mm)	18.55 (6.52 − 21.78)	17.09 (13.23 − 18.08)	0.394
Actual MSP (mm)	19.91 (5.83 − 21.21)	17.78 (11.49 − 20.4)	0.336
Absolute MSP Difference (mm)	0.64 (0.17 − 2.56)	1.71 (0.79 − 2.42)	0.0649
Planned FHP (mm)	32.66 ± 6.91	27.71 ± 4.81	0.184
Actual FHP (mm)	32.46 ± 6.71	26.94 ± 4.55	0.130
Absolute FHP Difference (mm)	0.52 (0.36 − 2.33)	0.42 (0.01 − 1.93)	0.521
Planned CP (mm)	20.75 ± 6.63	19.46 ± 5.13	0.714
Actual CP (mm)	20.11 ± 7.6	20.87 ± 5.57	0.847
Absolute CP Difference (mm)	1.07 ± 0.6	1.73 ± 1.51	0.3513

* Significant (*p*-value < 0.05.); MSP = mid-sagittal plane; FHP = Frankfort horizontal plane; CP=coronal plane

In contrast, apical point measurements relative to anatomical planes showed significantly greater absolute deviation in only apical MSP for Group II compared to Group I (*p* = 0.015). This indicates increased apical implant positioning variability with partially limiting guides (Table 4).

**Table 4:** Linear deviations at the apical point relative to the anatomical planes (CP, MSP, FHP) in the study groups

Variable	Group I (Complete Limiting)	Group II (Partial Limiting)	*p*-value
Planned MSP (mm)	54.43 (44.76 − 58.91)	48.36 (42.82 − 52.06)	0.132
Actual MSP (mm)	52.71 ± 4.94	49.18 ± 1.5	0.1456
Absolute MSP Difference (mm)	0.90 (0.11 − 2.06)	2.04 (1.47 − 5.95)	0.015*
Planned FHP (mm)	4.91 ± 3.42	6.25 ± 1.84	0.423
Actual FHP (mm)	4.26 ± 2.72	6.28 ± 1.766	0.161
Absolute FHP Difference (mm)	1.25 ± 0.56	1.40 ± 0.95	0.753
Planned CP (mm)	27.26 ± 2.41	25.13 ± 5.17	0.39
Actual CP (mm)	27.77 ± 4.17	25.98 ± 5.52	0.542
Absolute CP Difference (mm)	2.07 ± 1.04	2.21 ± 1.46	0.851

* Significant (*p*-value < 0.05.); MSP = mid-sagittal plane; FHP = Frankfort horizontal plane; CP=coronal plane

## Discussion

The ZI placement remains surgically challenging due to restricted visibility and complex zygomatic anatomy, including the curved sinus lateral wall and sinusoidal posterior wall. These factors complicate drilling precision and implant positioning, raising risks of orbital or infratemporal fossa penetration [3, 4]. Digitally guided surgery utilizes virtual planning data to create patient-specific templates that achieve accuracy, maintain critical anatomical safety zones, and deliver prosthetically optimal implant positions [17, 20–22]. The aim of this study was to compare the accuracy of completely limiting versus partially limiting computer-generated 3D surgical guides for zygomatic implant placement in patients with atrophic maxillae.

The ZAGA classification plays a crucial role in shaping the surgical guides used for zygomatic implant placement. Understanding this classification allows practitioners to tailor the surgical guide design to account for individual anatomical variations, thereby enhancing the accuracy and effectiveness of surgical procedures [13]. The ZAGA classification aids in the strategic planning for implant placement [9, 19, 23]. The ZAGA classification influences the design of surgical guides by defining the necessary drill pathways and stabilizing features for zygomatic implants. For ZAGA type 0 and type 1 cases, where bone volume is sufficient, simpler surgical guides can be utilized, allowing placement of implants through predictable intra-sinus pathways. Conversely, cases categorized as ZAGA type II and above, which may present anatomical complexities such as unfavorable maxillary sinus positioning or significant atrophy, necessitate more intricate surgical guides. These guides may integrate features like matching window osteotomies to safeguard the sinus membrane and enhance precision during zygomatic implant placement [13, 19, 23].

In this study, all participants were classified as ZAGA II–III. Accordingly, the surgical guide design incorporated a cut-off window aligned with the planned implant trajectory. Customized guides that accommodate the anatomical nuances identified by ZAGA can enhance accuracy in implant placement, thereby reducing the risk of complications like inadvertent sinus perforation and improving overall treatment outcomes [23–25]. Studies suggest that precision instruments adhering to ZAGA principles, such as double-sleeve guides, are vital in simplifying the surgical process and optimizing placement accuracy, which is essential for patients undergoing zygomatic implants [24]. Moreover, the emphasis on patient-specific anatomical factors, as guided by the ZAGA classification, underscores the importance of meticulous preoperative imaging and planning [9, 19, 23].

The accuracy of the ZI surgical guide critically depends on the stable positioning of the surgical template on the bone. In the current study, the surgical guide design incorporated maximum coverage of the exposed maxillary bone surfaces to achieve optimal stability, with particular attention to the zygomatic arch, palatine process, and anterior nasal spine, creating a biomechanically advantageous tripodal support system. This triangular distribution of contact points provided three-dimensional resistance to movement, effectively preventing displacement along any axis during the drilling sequence.

In the current study, post-zygomatic implant placement examinations revealed that all patients were free from clinical symptoms of sinus disruption, such as nasal congestion, nasal discharge, headaches, oroantral communication, or oroantral fistula. The absence of sinus-related complications in all post-operative examinations following ZI placement serves as a clinically significant indicator of procedural accuracy and surgical success. patients exhibited no evidence of sinus disruption manifestations which are established markers of iatrogenic trauma or mispositioned implants. This complication-free outcome profile validates the precision of the implant placement technique and underscores the clinical reliability of the surgical protocol employed. Such findings carry particular weight in zygomatic implantology, where the intimate anatomical relationship between implant trajectories and maxillary sinus structures makes the avoidance of sinonasal morbidity a key metric of procedural excellence. Studies have consistently reported that properly zygomatic implant placements, particularly those utilizing surgical guides, result in a favorable postoperative course without significant sinus complications [13, 19, 25]. Furthermore, Menchini-Fabris et al. [26] and Pérez et al. [27] reported that zygomatic implants have high success rates largely due to meticulous planning. This planning often employs computer-assisted techniques that enhance the precision of implant placements and can predict potential complications, thereby minimizing risks of postoperative symptoms.

This study demonstrated a statistically significant reduction in operative time when using partially limiting guides (21.17 ± 2.14 min) compared to complete limiting guides (27 ± 2.37 min; *p* = 0.01). The observed discrepancy may result from the increased technical difficulty associated with complete limiting surgical guides, including restricted drill movement through the metal sleeves and the greater influences of anatomical constraints such as limited mouth opening and interference from mandibular dentition. On the other hand, partially limiting guides provide mechanisms that establish certain controls over the drilling process while simultaneously allowing some degree of flexibility, which can lead to improved clinical outcomes.

The observed reduction in operative time, while statistically significant, averaged approximately six minutes per implant (a 15–20% relative decrease). The practical significance of this finding is context dependent. In high-volume surgical practices, this incremental gain can enhance daily workflow efficiency. For extensive full-arch reconstructions cases, cumulative time saving may also reduce patient chair time and procedural fatigue. It is important to contextualize this benefit within a broader assessment, as it must be offset against the greater financial investment and additional planning time required for fully guided static surgery. Moreover, the ability to decrease surgical time while employing a partially limiting guide can lead to quicker recovery for patients. This notion resonates with findings from Tallarico et al. [28], which indicated that improved accuracy and the use of less invasive techniques can reduce chair time and patient recovery time in dental implant surgeries, thus emphasizing the correlation between surgical guidance quality and overall patient care experience.

Results of this study confirmed a negligible difference between the two groups, as linear and angular deviations have shown no statistically significant differences. This analysis confirms a comparable accuracy between the two guiding protocols. The results of the linear and angular deviations fall within the acceptable ranges according to a study by Van Steenberghe et al. [20] that showed linear deviation below 3 mm and angular deviation below 3.5 degrees.

In this study, the coronal point measurements relative to the three anatomical planes demonstrated no statistically significant differences between the two groups for all coronal parameters. In contrast, apical point measurements relative to the anatomical planes revealed significantly greater absolute deviation in only the midsagittal plane (MSP) for Group II compared to Group I (*p* = 0.015). Notably, the statistically significant difference in apical deviation (*p* = 0.015) suggests that the fully guided protocol may provide a distinct advantage in achieving precision at the critical apical endpoint, particularly in the sagittal dimension. This finding aligns with a previous systematic scoping review, which demonstrates a more precise coronal entry point position on the alveolar ridge [17]. Additionally, Gercchi et al. [8] stated that most of the guide systems transfer accurate planned coronal positions but cannot transfer apical positions accurately due to limited access during surgery, flexibility of long drills, and curved or irregular bony surfaces at the base of the zygomatic bone. The observed increase in apical deviation in partially guided systems may result from a combination of biomechanical and procedural factors. While the guide accurately controls the initial coronal entry point, the subsequent freehand drilling phase introduces potential for error. Even minor angular inaccuracies at the coronal level are magnified along the long axis of the drill, leading to significantly larger positional discrepancies at the implant apex. Furthermore, partially limiting guides inherently offer less rigidity and allow for more drill flexibility within the osteotomy site. This reduced restraint, combined with the challenges of navigating long drills through complex anatomical pathways with limited visibility, contributes to increased variability in the final apical implant position. In contrast, fully guided systems provide rigid sleeves that constrain the drill throughout its entire path, minimizing angular deviation and effectively controlling both the coronal and apical endpoints [24, 29].

According to the present study, partially limiting guides provide mechanisms that establish certain controls over the drilling process while simultaneously allowing the surgeon some degree of flexibility, which can lead to improved clinical outcomes. The classification of surgical guides into partially limiting and completely limiting designs underscores the importance of tailored approaches based on specific clinical needs [13, 16, 17]. In instances where anatomical constraints or the demands of precise positioning preclude the use of a completely restricted drill path, partially guided protocols offer a strategic compromise. They provide a critical balance between surgical guidance and intraoperative adaptability, allowing clinicians to navigate complex spatial relationships while largely maintaining the intended implant trajectory. However, this adaptability entails a trade-off in predictability. Although the mean apical deviation observed in the partially guided group remained within clinically acceptable limits, the significantly greater variance indicates less consistent control at the critical apical implant point. In anatomically high-risk sites this reduced predictability could increase the risk of encroachment upon important vital structures. This inherent limitation underscores the clinical advantage of the fully guided protocol, where its statistically superior precision at the apical position enhances safety margins and offers a more predictable safeguard in regions of sensitive anatomy.

This study presents several limitations that warrant consideration when interpreting the results. Firstly, the small sample size inherently limits the statistical power and the generalizability of the findings. Therefore, the absence of statistically significant differences for several deviation parameters should be interpreted with caution, as a larger study might reveal differences that were not detected in this study. Secondly, the single center introduces a potential for operator bias and may not reflect the variability encountered in broader clinical practice. Thirdly, the short-term nature of the postoperative assessment provides data on initial accuracy but does not inform on long-term clinical outcomes, survival rates, or late-onset complications. Fourthly, in this study, although implants were analyzed as independent units, each patient received two zygomatic implants following a standardized treatment protocol, resulting in equal cluster sizes for all patients. This balanced distribution of cluster sizes could minimize the potential effect of intra‑patient correlation on variance estimation. However, future studies with larger sample sizes may consider multilevel statistical modeling to further account for clustering effects. Finally, while a statistically significant difference in apical deviation was noted in the midsagittal plane, the study is underpowered to conclusively establish clinical equivalence between the two guided protocols, particularly for apical positioning. These findings should therefore be viewed as preliminary, highlighting the need for further investigation.

Further studies with larger sample sizes are required to assess the predictability of partially limiting surgical guides on the survival rate and complication of zygomatic implants. Additionally, future research should explore hybrid solutions that integrate the precision of complete limiting guides with the adaptability and flexibility of partially limiting guide protocol.

## Conclusion

Within the limitations of the sample size of this study, the partially limiting surgical guide could provide a successful and less invasive treatment modality for zygomatic implant placement, as it significantly reduces the surgical time with comparable overall accuracy. However, the completely limiting guide demonstrates superior control at the critical apical implant position in the midsagittal plane. Further studies with a larger sample size are required to substantiate these observations and to formulate definitive clinical recommendations.

## Supplementary Information

Supplementary Material 1.

## Data Availability

The datasets used and/or analyzed during the current study are available from the corresponding author on reasonable request. For privacy reasons, however, individual data allowing for the identification of participants cannot be made available.

## Abbreviations

ZI: Zygomatic implants
CBCT: cone-beam computed tomography
DICOM: Digital Imaging and Communication in Medicine
STL: Standard Tessellation Language
ZAGA: Zygoma Anatomy Guided Approach
MSP: Mid-sagittal plane
FHP: Frankfort horizontal plane
CP: coronal plane
